# Obesogens: How They Are Identified and Molecular Mechanisms Underlying Their Action

**DOI:** 10.3389/fendo.2021.780888

**Published:** 2021-11-25

**Authors:** Nicole Mohajer, Chrislyn Y. Du, Christian Checkcinco, Bruce Blumberg

**Affiliations:** ^1^ Deparment of Pharmaceutical Sciences, University of California, Irvine, CA, United States; ^2^ Deparment of Developmental and Cell Biology, University of California, Irvine, CA, United States; ^3^ Deparment of Biomedical Engineering, University of California, Irvine, CA, United States

**Keywords:** EDC, MDC, obesity, endocrine disrupting chemical, obesogens, adipogenesis, metabolism disrupting chemicals

## Abstract

Adult and childhood obesity have reached pandemic level proportions. The idea that caloric excess and insufficient levels of physical activity leads to obesity is a commonly accepted answer for unwanted weight gain. This paradigm offers an inconclusive explanation as the world continually moves towards an unhealthier and heavier existence irrespective of energy balance. Endocrine disrupting chemicals (EDCs) are chemicals that resemble natural hormones and disrupt endocrine function by interfering with the body’s endogenous hormones. A subset of EDCs called obesogens have been found to cause metabolic disruptions such as increased fat storage, *in vivo*. Obesogens act on the metabolic system through multiple avenues and have been found to affect the homeostasis of a variety of systems such as the gut microbiome and adipose tissue functioning. Obesogenic compounds have been shown to cause metabolic disturbances later in life that can even pass into multiple future generations, post exposure. The rising rates of obesity and related metabolic disease are demanding increasing attention on chemical screening efforts and worldwide preventative strategies to keep the public and future generations safe. This review addresses the most current findings on known obesogens and their effects on the metabolic system, the mechanisms of action through which they act upon, and the screening efforts through which they were identified with. The interplay between obesogens, brown adipose tissue, and the gut microbiome are major topics that will be covered.

## Obesity Is a Serious Problem

Obesity has become a present-day pandemic affecting people of all ages across the world. According to the World Health Organization, the prevalence of global obesity has nearly tripled since 1975 with a continued upward trajectory (1). In 2016, the WHO reported more than 1.9 billion adults as overweight, with 650 million of those adults as obese. The prevalence of obesity in children has continued to rise in the U.S alone, despite the nation’s efforts to promote better nutrition practices and increase physical activity levels in the educational system (2). In 2019, a staggering 38.2 million children under the age of 5 were reported as overweight or obese, worldwide (1). Comorbidities associated with obesity affect nearly all physiological systems and lead to serious health complications including mortality and a lowered quality of life. Obesity contributes to a growing list of health complications including insulin resistance, cardiovascular diseases, airway dysfunctions, metabolic syndrome, kidney disease, osteoarthritis, skin diseases, reproductive disorders, and cancer (3, 4) and death from COVID-19 (5). In addition to physiological comorbidities, the burden of obesity affects the individual’s psychological well-being, leading to higher stress and depression. Obesity often presents together with depression and negative self-image in both children and adults, creating a vicious cycle where the conditions potentiate each other (3, 6). Those who suffer from depression are 58% more likely to develop obesity, and those who are obese are 55% more likely to develop chronic depression (7). Obesity makes it less likely for students to stay in school past the 12^th^ grade, independently of their parent’s socioeconomic status (8). Similarly, lower education levels have been linked to higher weight gain and obesity (9). Obesity places a financial and emotional burden on individuals, their families, and the nation at large when loss of productivity and loss of work is considered. The CDC reported the national obesity-related cost to be $147 billion in 2008, however, more recent data from 2014 estimates the cost of obesity and its comorbidities to be closer to $2 trillion dollars (10, 11). It is estimated that the annual cost of obesity in the U.S will rise $48-66 billion each year throughout 2030 (4). Therefore, the severe consequences of obesity on both individual and population-level health demand that urgent attention be paid to this worsening pandemic.

## Obesity Is More Than Calories In/Out

Obesity is a multifaceted disease, and its etiology remains widely misunderstood. Weight gain has primarily been blamed on high calorie diet and a sedentary lifestyle. Many types of fad diets have shown short-lived improvements in body weight, but the overall success for long-term weight loss through caloric restriction remains inefficacious and the global prevalence of obesity continues to rise. Recent research has highlighted the shortcomings of the energy balance, or “calories in versus calories out” paradigm of weight management. The idea that people must consume less calories than they burn in order to lose weight is self-evident, but is no longer an all-inclusive explanation for the increasing rate of obesity and long-term weight gain.

Some lower income countries have reported a decrease in exercise, other higher income countries, such as the U.S., have reported a consistent or even increased level of exercise over the last 30 years despite the continuous rise in obesity (12, 13). If the population is gaining weight despite recommended physical activity levels, then the problem must also include the nature of the foods ingested as well as energy expenditure. We must consider the *quality* of the calorie being consumed since not all calories are created equal. The quality of the calorie, and whether it is coming from healthy foods or unhealthy foods, influences the types of food we should and shouldn’t eat and how our bodies metabolize those calories for maximal benefit. Primates who were given calorically equal meals that only differed in the percentage of cis or trans-fats showed a disparity in weight gain after six years, with the trans-fat group showing an increase in visceral fat (14). The composition of our diets, more so than the caloric count of our daily diets, affects hormonal imbalances, metabolic efficiency, epigenetics, gut health, and fat accumulation (15). According to the carbohydrate-insulin model of obesity, the way we metabolize processed carbohydrates and foods that are higher on the glycemic index (such as starchy, refined, and sugary foods) promotes fat storage in fat cells and is driven by spikes in insulin levels (16). Therefore, eating the same number of calories in candy vs Brussel sprouts will be processed, metabolized, and stored in very different ways. Taken together, the current caloric models of obesity and weight gain are insufficient as stand-alone explanations for the sudden increase in global obesity over the past few decades.

## EDCs and Obesogens

While environmental, nutritional, and socioeconomic factors may all contribute to weight gain, there are other components of our immediate environment that offer a more in-depth explanation for the etiopathology of obesity. Increasing evidence has linked chemical exposure, ingestion, and inhalation of industrial compounds to obesity and other metabolic and endocrine related diseases. As the world modernizes, more chemicals pollute our food, water, air, and soil, making exposure unavoidable. Endocrine disrupting chemicals (EDCs) are chemicals or mixtures that disrupt endocrine function and interfere with the body’s endogenous hormones (17). EDCs are structured like and act similarly to natural hormones and disrupt homeostasis by binding to hormone receptors (18). Naturally found EDC’s include plant phytoestrogens such as those in soy-based foods and dairy products. Phytoestrogens can behave as endocrine disruptors by affecting estrogen receptor-mediated pathways (19). Synthetic EDCs are found in common industrial products such as pesticides, fungicides, flame retardants, plastics, food wrappers, solvents, and metals. Both *in vitro* and *in vivo* studies have shown that synthetic EDCs exert effects on multiple systems including the reproductive system, the central nervous system, the immune system, and on metabolic function (20). In addition to endocrine pathways there are many non-hormonal cellular signaling pathways that could potentially be disrupted by chemical exposures. The concept of “signal toxicity” has been developed to account for this potential disruption of the thousands of cellular signaling pathways that could be targeted (21). Relevant examples of signal toxicity include disruption of neurotransmitter signaling, growth factor signaling pathways, receptor kinase signaling, etc. These should not be ignored in the developing discussion about environmental chemicals and obesity.

While EDCs can affect multiple physiological systems, recent research has placed much needed focus on chemicals that might be associated with the rising rates of metabolic syndrome and obesity. A subset of EDCs act as obesogens – chemicals that lead to increased fat storage, *in vivo* after exposure [reviewed in (22–24)]. The environmental obesogen model proposes that obesogens cause greater susceptibility to weight gain, lipid storage, and energy imbalances that lead to obesity (25). In 2015, the Parma consensus broadened the definition of obesogens to include EDCs that affect other obesity related metabolic conditions that drive metabolic syndrome, such as insulin resistance, hypertension, dyslipidemia, and hyperglycemia (26). This class of EDCs was denoted as metabolism disrupting chemicals (MDCs) [reviewed in (26, 27)]. Many chemical obesogens have been identified and numerous reviews have been written about them in recent years (22–24, 28). The total number of obesogens is currently unknown because no systematic attempt has been undertaken to identify them. This review will identify recent findings on possible obesogens, their effects on metabolism and lipid dysregulation and the mechanisms through which they act.

## Adipogenesis, Nuclear Receptors, and Key Pathways

The obesogen hypothesis holds that exposure to obesogenic chemicals will lead to increased white adipose tissue (WAT) mass. Adipogenesis is the cellular process by which pluripotent stem cells or preadipocytes commit their fate to differentiating into adipocytes (29). WAT can be found subcutaneously or viscerally, and too much WAT can result in in excess lipid storage, altered adipocyte homeostasis, the disruption of energy balance, and changes in metabolic set points [reviewed in (30)]. In healthy individuals, WAT plays an important role in metabolism and energy homeostasis throughout the body. However, people with obesity and type two diabetes (T2D) experience an inflammatory response in their adipose tissue, particularly in visceral white fat that contains higher levels of reactive oxidative species (29, 31). WAT differentiation and cell functioning is primarily controlled by the peroxisome proliferator-activated receptor gamma (PPARγ), also known as the “master” regulator of adipogenesis (32). PPARγ is a ligand-activated transcription factor which is responsible for the growth and development of adipose tissue and acts as the receptor for antidiabetic drugs such as rosiglitazone (32). Some obesogenic EDCs can bind to PPARγ, creating downstream effects that influence multipotent mesenchymal stromal stem cells (MSCs) to favor the adipogenic pathway. EDCs can bind to other nuclear receptors as well, including estrogen, androgen, and progesterone receptors, thyroid receptors, and retinoid X receptors (18). EDC action is not limited to nuclear receptors; EDCs can also bind to nonnuclear receptors and nonsteroid receptors (18). Orphan nuclear receptors, such as estrogen related receptor alpha and estrogen related receptor beta, have been found to play a role in metabolic disease, weight gain, and obesity when exposed to EDCs such as Bisphenol AF (18, 33, 34). When EDCs bind receptors that are meant to regulate vital cellular functions and cell signaling, major health consequences can arise, disrupting homeostatic mechanisms and correct development.

## *in Vitro* Assays for Obesogens

There are many *in vitro* models that can be used to assess the potential obesogenic properties of chemicals. A list of *in vitro* model systems and obesogens identified using these models is presented in **Table 1**. When using non-human cell types as obesity models, it is important to understand that the translational application to humans might sometimes be limited by differences between species (53). The first studies on adipogenesis and obesogens occurred in the early 2000’s on mouse 3T3-L1 cells derived from 3T3 cells [reviewed in (30)]. 3T3-L1 is a well-established preadipocyte cell line derived from 17–19-day old mouse embryos and has a fibroblastic morphology that can be readily induced into adipocyte differentiation (54, 55). To differentiate 3T3-L1 cells into adipocytes, the cells were treated with a minimum level of an adipogenic cocktail that often includes insulin, dexamethasone, and 3-isobutyl-1-methylxanthine (56). The benefits of this cell type include its ease of culture and cost effectiveness compared to mature adipocytes and other primary cells. 3T3-L1 cells have been used for toxicogenomic studies aimed at evaluating the efficacy of screening for obesogens (57). Although these cells can maintain high stability in transcription patterns, they produce differing lipid accumulation levels between tested compounds which can interfere with the interpretation of the mechanistic possibilities (57). 3T3-L1 cells are also highly sensitive to small perturbations in assay conditions such as brand of plastic plates, batches of bovine sera, origin and passage number of cells, and density at induction, all of which can impact their utility (58).

**Table 1:** *In vitro* model systems and associated obesogens.

Model System(*in vitro*)	Chemical	Source/Use	Proposed Mechanism	Effects	References
3T3-L1	3-tertbutyl-4-hydroxyanisole (3-BHA)	Used anthropogenic antioxidants in food	Regulated transcriptional and protein levels of the adipogenetic biomarkers upstream of the PPARγ signaling pathway	Induces the differentiation of adipocytes and increases cellular lipid accumulation	(35)
	Bisphenol A (BPA)	Used in personal products, household care products, and plastics	PPARγ activator	Induces the differentiation of adipocytes	(36, 37)
	Parabens	Used as cosmetic preservatives and as bactericides/fungicides	PPARγ activator	Induces the differentiation of adipocytes	(36)
	Phthalates	Used in cosmetics, pharmaceuticals, paints, medical equipment, and plastics	PPARγ activator	Induces the differentiation of adipocytes	(36)
	Tonalide	A musk compound used as a synthetic perfume	Acts *via* non-PPARγ mediated mechanism; more research needed.	Induces the differentiation of adipocytes	(36)
	Bisphenol A diglycidyl ether (BADGE)	Used in the manufacturing of coatings and resins	Proposed to act through a mechanism that is downstream of/parallel to, PPARγ.	Induces adipogenesis	(38)
	Bisphenol S (BPS)	Used as a substitute for BPA in plastics	Targets the PGC1α and the ERRγ genes	Increases cellular lipid accumulation, increases glucose uptake, and increases and leptin production	(39)
	Dibutyltin (DBT)	Used to manufacture products containing plastic and rubber materials	PPARγ/RXRα partial activator	Induces adipogenesis	(40, 41)
	Triphenyltin	Fungicide/antifoulant	PPARγ and RXRα activator	Stimulates adipocyte differentiation and increases the expression of adipocyte marker genes	(42)
	Dioctyl sodium sulfosuccinate (DOSS)	A major component of the oil dispersant, COREXIT; widely used in pharmaceuticals, flavored drinks, and personal care products	PPARγ activator	Induces adipogenesis and increases cellular lipid accumulation	(43)
	Imidacloprid	Insecticide	Proposed to be mediated *via* the pregnane X receptor	Increases adipocyte differentiation and lipogenesis	(44, 45)
	Mono-Ethylhexyl Phthalate (MEHP)	Used in manufacturing products made of polyvinyl chloride	PPARγ and PPARα activator	Increases adipocyte differentiation and insulin sensitization	(46)
	Quizalofop-p-ethyl	Pesticide	PPARγ activator	Induces lipid accumulation	(47)
	Sorbitan monooleate (Span 80)	A nonionic surfactant and a component of Corexit	Transactivates RXRα	Promotes adipogenesis	(48)
	Tributyltin (TBT)	Biocide/antifoulant/pesticide	PPARγ and RXRα agonist	Induces adipogenesis, increases triglyceride storage, and increases the expression of adipogenic marker genes	(42, 49, 50)
OP9	Pioglitazone	Used in pharmaceuticals	PPARγ agonist	Enhances lipid accumulation	(51)
	Prallethrin	Insecticide	PPARγ agonist	Enhances lipid accumulation	(51)
Human embryonic derived stem cell (hESC’s)	Bisphenol A (BPA), Bisphenol S (BPS)	Found in plastic products	PPARγ agonist	Increases triglyceride levels and increases expression of adipogenic genes	(52)

Another cell type that has been used for a similar purpose is the C3H10T½ cell line, which was developed in 1973 from mouse embryonic stem cells (59). These cells can differentiate into various mesodermal cell types including myocytes, chondrocytes, and adipocytes. C3H10T½ cells exhibit a fibroblast like morphology resembling multipotent MSCs, leading some investigators to mistakenly refer to these cells as bona fide MSCs. The primary applications for C3H10T½ cells have centered around evaluating the impact of compounds on adipogenesis and the molecular mechanisms underlying adipogenic differentiation (60). Notable characteristics of these cells are their ability to maintain a relatively homogenous population of multipotent stem cell-like cells and their usefulness in assessing adipocyte commitment and differentiation.

The OP9 mouse stromal cell line was developed from the calvaria of newborn mice that were genetically modified to be deficient in macrophage colony stimulating factor. This bone marrow derived stromal cell line is known for its ability to collect large amounts of triglyceride droplets when stimulated towards adipogenesis after 72 hours, allowing these cells to be an appropriate model for rapidly screening chemicals for adipogenic effects (61, 62). A clonal derivative denoted as OP9-K cells was later developed that could differentiate rapidly and reproducibly. OP9-K cells are readily transfected with an efficiency of ~80% and were validated as a model system for microarray analysis of the differentiated transcriptome (63). In comparison with 3T3-L1 and mouse bone-marrow derived MSCs, OP9 cells were more sensitive to the induction of adipogenesis by chemicals known to activate PPARγ and RXR (51, 58). The OP9 cell line appears to be a promising *in vitro* model to study adipogenesis using mouse cell lines.

Multipotent mesenchymal stromal stem cells, also known mesenchymal stem cells (hMSCs) have been used to assess possible metabolic disruptors *in vitro* (64, 65). MSCs are used as an alternative to human pre-adipocytes which have reduced proliferative ability and can exhibit physiological differences related to the fat depot of origin within the body (66). MSCs are bona fide, multipotent precursors of a variety of cell types including adipocytes, chondroblasts, osteoblasts, and hematopoietic‐supporting stromal cells (67). MSCs can simultaneously express genes characteristic of various mesenchymal cell lineages while also maintaining osteogenic and adipogenic potential *in vitro* (68). A key advantage of the MSC model is that two important parameters of adipogenesis can be evaluated: commitment of stem cells to pre-adipocytes and the differentiation of pre-adipocytes to mature adipocytes (69, 70). The ability to evaluate both endpoints make MSCs a favorable system to study adipogenesis, although they are currently less utilized than the preadipocyte models such as 3T3-L1 cells. Using human MSCs may aid in understanding the effects of contaminants in humans as well as facilitate translational efforts (65).

## *In Vivo* Assays for Obesogens

*In vivo* models allow the study of chemical effects on complex organisms in which multiple systems must work simultaneously in a natural physiological environment. Using *in vivo* models is a valuable tool in determining if and how chemicals act as EDCs in a way that is more translatable to how EDCs may act in humans. Various *in vivo* model systems and obesogens identified using these models are listed in **Table 2**. Rodents, particularly mice have been a very widely used model to study the effects of chemical exposures and infer possible effects in humans. Mouse models have allowed more focus to be placed on the developmental origins of disease, which aids in understanding the origins of chronic and adult-onset diseases. Mouse models have also allowed for the discovery of obesogens and endocrine disruptors.

**Table 2:** *In vivo* model systems and associated obesogens.

Model System*(in vivo*)	Chemical	Source/Use	Proposed Mechanism	Effects	References
Mice	Tributyltin (TBT)	Biocide/antifoulant/pesticide	PPARγ and RXRα agonist	Increases epididymal adipose mass in adults. Increases lipid accumulation in adipose depots, liver, and testis of neonate mice.	(49)
	Bisphenol A (BPA)	Found in plastic products such as water pipes and toys; found in electronic equipment	Acts partially through GR signaling; enhances expression of adipogenic genes and lipogenic enzymes, acts on PPARγ	Increases body weight, fat mass, chronic inflammation, and inflammation in white adipose tissues.	(71, 72)
	Bisphenol S (BPS)	A BPA substitute; used in the manufacture of plastics and resins. Ingestion from food is the major source of BPS exposure	PPARγ activator; increases expression of PPARγ	Increases liver triglycerides, causes hyperinsulinemia, induces changes in gene expression, causes changes in liver DNA methylation.	(73, 74)
	Diethylstilbestrol (DES)	A synthetic estrogen previously used in pharmaceuticals during pregnancy	Estrogenic activity	Induces significant increase in body weight and reproductive abnormalities	(75, 76)
	Dichlorodiphenyltrichloroethane (DDT) and dichlorodiphenyldichloroethylene (DDE)	Pesticide DDE is the metabolite of DDT	Inconclusive	Induces thermogenic impairment of brown adipose tissue, obesity, insulin resistance, and dyslipidemia	(77, 78)
	DBT	Used to make of polyvinyl chloride (PVC) plastics and medical devices	PPARγ and RXRα agonist; increases the expression of adipogenic genes	Induces increased lipid accumulation, fat storage, leptin levels, and glucose intolerance.	(79)
	Triflumizole	Fungicide	PPARγ activator	Increases adipose depot weight and adipogenic gene expression	(80)
	Tolylfluanid	Fungicide	Acts through glucocorticoid receptor signaling	Induces higher body weight, fat mass, visceral adipose depots, glucose intolerance, insulin resistance, and metabolic and energy disturbances	(81)
	Diethyl-hexyl-phthalate (DEHP)	Found in personal care products, lubricants, pesticides, paints, and PVC plastics. Exposure is mainly through food *via* food packaging	PPARγ activator	Increases body weight, adipose tissue, lipids, and glucose levels	(82)
	Cadmium (Cd)	Ingestion of contaminated foods	Inconclusive	Induces metabolic syndrome-like phenotypes (impaired glucose and insulin functioning, hepatic steatosis, weight gain, increase in fat), oxidative stress and mitochondrial dysfunction.	(83)
	Di (2-ethylhexyl) phthalate	Used in the making of PVC plastics and vinyl products; used in lubricants, emulsifying agents, and cosmetics	Possible PPARγ activator	Induces glucose intolerance, insulin resistance, hepatic steatosis/steatohepatitis, increased leptin levels, increased cholesterol, and white adipose tissue disfunction.	(84)
Rats	Bisphenol A (BPA)	Found in plastic products	Activates Erα and Erβ; thyroid hormone receptor antagonist	Induces an increase in body weight and white adipose tissue, adipocyte hypertrophy, and increased expression of adipogenic genes	(85)
	Tributyltin (TBT)	Biocide and molluscicide	RXR and PPARγ activation	Causes ovarian obesogenic effects	(86)
Zebrafish (*Danio rerio*	Mono ethyl phthalate (MEHP) and	Primary metabolite of di(2-ethylhexyl) phthalate (DEHP)	PPARγ agonist	Obesogenic properties	(87)
	Tetrabromobisphenol A (TBBPA)	Flame retardant	PPARγ agonist	Obesogenic properties	(87)
	Cadmium	Ingestion of contaminated foods, cigarette smoke, and breathing contaminated air	Inconclusive	Increased lipid accumulation	(88)
Frog (*Xenopus laevi)*	Tributyltin (TBT)	Biocides, antifoulants, pesticides	PPARγ and RXRα agonist	Formation of ectopic adipocytes in and around gonadal tissues	(49)

Diethylstilbestrol (DES), a chemical that was once prescribed to prevent miscarriage, was discovered to be a dangerous EDC that causes lifelong health issues (75). DES was identified as a chemical that increased adiposity, *in vivo*, presumably as a result of its action on the estrogen receptor (76).

Mouse models also made it possible to show that tributyltin chloride (TBT) not only caused adipocyte differentiation in cell models but had adipogenic effects *in vivo*. TBT studies began with *in vitro* models using 3T3-L1 cell lines and were later extended into animal models. Triphenyltin and TBT were discovered to be activators of PPARγ and retinoid X receptor (RXR) *via* a preadipocyte 3T3-L1 cell model (42). A contemporaneous study showed that TBT could elicit adipocyte differentiation in 3T3-L1 cells and fat accumulation in mice treated prenatally (49). Dibutyltin (DBT) is an organotin used as a heat stabilizer in polyvinyl chloride (PVC) plastics and is also a metabolite of TBT. DBT was shown to be a PPARγ and RXR activator, *in vitro*, and inducer of adipogenesis in 3T3-L1 cells and in human and mouse MSCs (40, 41). Perinatal exposure of pregnant C57BL6/J mouse dams led to increased leptin levels, glucose intolerance, and increased fat storage in adulthood, confirming DBT’s obesogenic effects in a complex organism (40). Common fungicides have also become classified as obesogenic EDCs after being screened using mouse models. Triflumizole was found to be a PPARγ activator *in vitro*, and when tested in gestating female mice, it was found to increase adipose depot weight and shift MSC fate to favor adipogenesis (80). Another common fungicide, tolylfluanid, was shown to be a glucocorticoid receptor activator *in vitro*, and obesogen in mice (81).

Rodent studies have been used to confirm the obesogenic action of a number of plastic monomers, plasticizers and other additives [reviewed in 27](**Table 2**). More recent studies testing the safety of BPS, a BPA substitute used in the manufacturing of plastics, have used *in vivo* mouse models to determine if BPS exhibits obesogenic properties by affecting gene expression and DNA methylation. It was found, for the first time, that even low doses of BPS acted as an *in vivo* obesogen and caused epigenetic changes in genes related to metabolism (73). BPA and BPS were found to target PPARγ in human macrophages and were confirmed to cause metabolic abnormalities through PPARγ in mouse models (74). Heavy metal exposure was shown to be obesogenic in mice [reviewed in 27]. CD-1 female mice that were exposed to cadmium *via* their drinking water from gestation to postnatal day 10 showed delayed obesogenic properties in female offspring, revealing that cadmium exposure can contribute to obesity later in life (83). This has particular relevance for populations living in the desert southwest of the US where heavy metal exposure *via* dust is prevalent.

It is important to note that some of the effects of early life obesogen exposure can be transmitted to future generations. When adult male or female animals are exposed to a chemical, they (the F0) are exposed as are germ cells within the animals (F1) generation. Effects observed in the F2 generation and beyond are considered transgenerational because these generations were not exposed to the chemical (89). In contrast, exposure to a gestating female mammal will elicit direct effects in the F0 (adult), F1 (embryo) and F2 (germ cells in the embryo) generations. Therefore, the F3 generation is the first not to be exposed and effects seen in F3 and beyond are transgenerational. This topic has been reviewed extensively in recent years and will not be discussed further here for brevity (23, 90–92).

While rodents have been the primary model used to study EDCs, less complex organisms have also proven to be valuable models in studying chemical exposure (**Table 2**). The adipogenic pathways taking place in less complex organisms also show interspecies similarities to those in higher organisms while the assays themselves are less expensive, shorter, and higher throughput. Zebrafish larvae have been used as an exposure model to test the effects of obesogen exposure on lipid accumulation *via* Oil red-O staining and to activate PPARγ. Test compounds such as the halogenated BPA analog tetrabromobisphenol-A (TBBPA) were found to induce zebrafish larval lipid accumulation (93). Exposure to environmentally relevant TBT concentrations resulted in adipocyte hypertrophy within only hours after exposure in zebrafish larvae, demonstrating the potency of TBT toward adipogenic endpoints (94).

Other species have also been used to screen for potential obesogens *in vivo*, such as the African clawed frog, *Xenopus laevis* (**Table 2**). X. laevis larvae were used to create a PPARγ reporter model to compare the metabolic capacity to those in mammals. A transgenic approach was used to express both human PPARγ and a series of PPARγ Response Element (PPRE)-eGFP reporter genes simultaneously (87). This approach enabled the detection of PPARγ activators using an *in vivo* context.

The effects of MDCs can even be studied using invertebrate models to further assess the underlying mechanisms through which chemicals alter lipid homeostasis. These included species like the fruit fly, *Drosophila melanogaster*, the water flea, *Daphnia magna*, and the roundworm, *Caenorhabditis elegans.* Invertebrates possess organ systems that allow nutrient uptake, storage, and energy metabolism through forms like glycogen and lipids (95). Since many intermediary pathways of metabolism are conserved, invertebrates can be a valuable model system for measuring metabolic change. For example, exposure of *D. melanogaster* to the plasticizer, dibutyl phthalate (DBP) led to increased lipid storage, starvation resistance, hyperglycemia, and hyperphagia in males *via* evolutionarily conserved insulin and glucagon-like signaling pathways (96). Long term parental exposure of *D. melanogaster* to a typical plasticizer, Bis(2-ethylhexyl) phthalate (DEHP), elicited significant change in body weight of offspring. The specific changes depended on the exposure period, dose, and gender of the exposed parent. Paternal DEHP treatment resulted in increased body weight of male offspring, whereas maternal exposure led to weight loss in male offspring (97). Exposure of a variety of aquatic invertebrates (coral, rotifers, copepods, octopus, scallop, crab, urchins, and worms) to environmental chemicals (PBDEs, phthalates, organotins or nanoparticles) led to alterations in the expression of genes important for *de novo* lipogenesis, fatty acid modification and triacylglycerol synthesis (98). *BDE-47 increased de novo lipogenesis in the copepod, Tigriopus japonicus after only 24 hours of exposure* (99)*. Exposure of the water flea, Daphnia magna to known mammalian obesogens including TBT and BPA enhanced fat storage, whereas exposure to DEHP or triphenyltin* impaired growth and reduced fat storage (100). Experiments such as these confirm the value of less complex model organisms to identify conserved mechanisms underlying metabolic disease.

## Thermogenic Adipocytes and EDCs

Broadly speaking, there are two major types of adipose tissue found in the human body: white adipose tissue (WAT), which primarily stores lipids and is maintained throughout adulthood, and thermogenic brown adipose tissue (BAT) which “burns” lipids and is primarily found in newborns and infants. BAT was previously thought to be non-existent or very minimal in adult humans but there are indeed brown fat depots found in adults (101). CT and PET-CT scans revealed multiple locations of BAT within the adult body. Brown adipose tissue is found both subcutaneously and viscerally. The major BAT depot in adult humans is subcutaneous in the supraclavicular region with smaller deposits under the clavicles and in the axilla (102). Visceral BAT can be perivascular, perivisceral and around solid organs such as the pancreas, kidney, liver and spleen (102). White adipocytes make up most of our body fat mass and form the visceral and subcutaneous fat tissues that store energy in the form of triglycerides. White adipose cells typically contain a large unilocular lipid droplet while brown adipocytes contain smaller, multilocular droplets that are rich in mitochondria (103). Brown adipocytes exhibit thermogenic activity when uncoupling proteins such as UCP1 are activated in response to environmental stimuli, mainly exposure to cold temperatures (104). Due to their large number of mitochondria, brown adipocytes act as energy generators rather than energy storers and burn calories as heat is expended. In addition to these distinct types of fat cells, a third, hybrid type of fat known as beige or brite adipose tissue, can form past infancy into adulthood. This process, known as “browning” or “beiging”, occurs as WAT is exposed to stimuli such as cold temperatures, catecholamines, physical activity, or thiazolidinediones, transforming them into brown-like, mitochondria rich, thermogenic adipocytes (105).

The discovery that brown and beige adipocytes exist in adults has raised increased interest in possible therapeutic strategies to treat obesity and type 2 diabetes (T2D) through the browning of white adipose tissue. Abundance of brown adipose tissue is associated with lower levels of metabolic disorders such as T2D, and its presence is associated with the improvement of insulin resistance (106). Increased BAT resulting from 10 days of cold exposure in 14-15°C resulted in a 43% increase in insulin sensitivity in people with T2D (107). Enhancing the formation or function of thermogenic adipocytes appears to be a promising key in the future treatment of obesity and related metabolic diseases (108, 109).

Since brown and beige adipose tissues are important for preventing obesity and T2D, interest is growing in the effects of EDCs on the formation and function of these valuable forms of thermogenic fat cells (110). Below we discuss some recent findings in this area. A list of obesogens associated with the disruption of thermogenic fat and adipose tissue is presented in **Table 3**.

**Table 3:** Chemical obesogens and their effects on thermogenic fat and adipose tissue.

Chemical	Source/Use	Proposed Mechanism	Effects	References
Bisphenols (A, F, S)	Chemical used to make polycarbonate plastics and epoxy resins. Found in the lining of food packaging.	Acts as an estrogen receptor agonist androgen, receptor antagonist	Shifts mesenchymal stem cell commitment and differentiation towards adipogenesis	(111, 112)
Dichlorodiphenyltrichloroethane (DDT) & dichlorodiphenyldichloroethylene (DDE)	Found in pesticides. DDE is a metabolite of DDT.	Acts as an estrogen receptor agonist, androgen receptor antagonist.	Induces a loss of BAT thermogenesis and affects the SNS that innervates BAT and WAT.	(77, 111, 113)
Silver nanoparticles (AgNPs)	Bactericides, found in fabric of athletic clothing to reduce odor.	Elevates the reactive oxidative species (ROS) levels within beige adipocytes and activates the MAPK-ERK signaling pathway.	Inhibits beige adipocyte differentiation, adipocyte thermogenesis, and mitochondrial functioning.	(114)
Arsenic	Polluted ground water	Lowers the expression of PPARγ, UCP1 and PGC1. Activates Estrogen Receptor	Inhibits the differentiation of BAT.	(115, 116)
Arsenite	A form of arsenic found in polluted water	Reduces UCP1 expression, accumulates in BAT, and suppresses Sestrin2 phosphorylation by ULK1. Activates Estrogen Receptor	Reduces BAT differentiation, decrease mitochondrial functioning, and lowers thermogenesis in BAT	(116–118)
Cadmium (Cd)	Released through the burning of fossil fuels. Used in electroplating, battery production, fertilizers.	Alters the gene expression of MCP-1 in WAT. Acts as an estrogen receptor agonist.	Elicits pro-inflammatory and carcinogenic effects. Causes damage to the kidneys, liver, lung, pancreas, testis, placenta, and bone. Causes metabolic disease including obesity and diabetes.	(119, 120)
Dechlorane Plus (DP)	Flame retardant	Downregulates UCP1 expression in BAT. Activates PPARγ pathway as an agonist.	Shifts BAT functioning towards that of WAT in a process termed “whitening” of brown adipocytes. Causes cells to be more prone to death, disrupts mitochondrial functioning, activates an inflammatory response by the accumulating macrophages around dead cells within WAT.	(121, 122)
Tetrabromobisphenol A (*TBBPA*) and analogs (TBBPA-MAE, TBBPA-MDBPE, TBBPA-BAE, AND TBBPA-BDBPE)	Flame retardant; found in plastic, and electrical equipment.	PPARγ and glucocorticoid receptor (GR) agonist	Causes adipogenesis. Increases lipid droplets in in hMSCs that differentiate into osteoblasts.	(123–125)
Dibutyl phthalate (DBP)	Found in plastic products including toothbrushes and food wrappers. Found in common household items are scented.	Estrogen receptor and PPARγ activator Activates CAR, PXR, PPARα, -β, and -γ).	Causes insulin resistance, increase in WAT, and endoplasmic reticulum stress.	(5, 126)
β-Cypermethrin (βCYP)	Used in insecticides		Promotes adipogenesis in stem cells. Increases ROS within cells by binding to the mitochondrial respiration chain complex 1.	(127)

## Bisphenols

The potential of BPA and its analogs as obesogens and their effects on brown thermogenic fat have become a topic of recent interest since many products labeled as “BPA free” likely contain its analog substitutes BPS and BPF. These compounds are found in products containing polycarbonate plastics and epoxy resins (including adhesives, plastics, paint, and sealants). BPS and BPF were shown to be as hormonally active as BPA, acting as estrogen receptor agonists and androgen receptor antagonists *in vitro* and *in vivo* [reviewed in (128)]. As obesogens, BPS and BPF elicited adipogenic differentiation in mouse preadipocytes and promoted the proliferation of fat cells, causing an increase in body mass (129). BPS and BPF exhibited obesogenic effects on human adipose-derived mesenchymal stromal stem cells (hADSC) in a dose dependent manner ranging from 0.01 to 25 μM (130). Pregnant F0 C57BL6/J mouse dams were exposed to a human-equivalent dose of BPS (1.5 μg/kg bw/day) throughout pregnancy and lactation. F1 pups were fed a high fat diet (HFD) over the next 15 weeks. Body weight was monitored weekly and body fat measured at euthanasia. Similar analyses were performed on the F2 and F3 generation offspring (131). Findings revealed interesting sex-dependent multigenerational effects, with multigenerational obesogenic effects found in both males and females, yet transgenerational effects only found in females, indicating that BPS is a likely sex-dependent obesogen. More discussion about differing effects on male and females can be found under sexual dimorphisms. Interestingly, BPA, BPS, and BPF have all been linked to a downregulation of the gene encoding micro RNA 26 (miR-26a) from *in vitro* studies. MiR-26a and miR-26b were shown to be key genetic regulators of the adipocyte browning process (132, 133). Both miR-26a and b are critical for adipogenesis and promote cellular pathways involving energy expenditure, mitochondrial formation, and the upregulation of uncoupling protein-1 (UCP-1) perhaps the key protein in thermogenesis (133).

## Dichlorodiphenyltrichloroethane and Dichlorodiphenyldichloroethylene

Dichlorodiphenyltrichloroethane (DDT) and dichlorodiphenyl-dichloroethylene (DDE) are two widespread organochlorine compounds that have been shown to affect thermogenic BAT and WAT in mouse models (134). DDT is a pesticide that is now banned in the United States, however, it remains in use in other parts of the world as a mechanism to control the spread of malaria (113). Both are known to collect in adipose tissue due to their lipophilicity. DDT and its metabolites such as DDE bioaccumulate up the food chain. DDT and DDE act as nuclear estrogen receptor agonists (DDT), androgen receptor antagonist (DDE), and can also bind to certain GPCRs, which can cause alter estrogen signaling (111). DDT has been shown in mouse models to affect the sympathetic nervous system that innervates both BAT and WAT. The sympathetic nervous system controls thermogenesis in brown fat and is needed for the beiging of WAT (77, 135). Dual administration of DDT and DDE or single treatment with DDE induced a loss of thermogenic abilities in BAT in adult female mice by reducing their BAT sympathetic innervation and regulation (77).

## Dechlorane Plus

The polychlorinated flame retardant, Dechlorane Plus (DP), is now known to impair BAT functioning and metabolic processes. DP has been found in humans in levels from 0.1 μg/kg to 1000 μg/kg, most concerningly in breast milk and umbilical cord serum (121). DP is a known PPARγ agonist, however, recent studies have shown that DP may also act *via* other pathways. DP exposure *in vitro* has shown an upregulation of adipogenic markers in the presence of a PPARγ antagonist, suggesting there could be an alternate pathway that DP may be acting on, independent of PPARγ (121). DP disrupted the function of BAT by down-regulating the expression of UCP1 mRNA, increasing lipid accumulation and disrupting mitochondrial functioning (121). These data were interpreted to indicate that BAT had been “whitened” (121). The whitening of BAT caused the affected cells to become more prone to cell death and activated an inflammatory response *via* the accumulation of macrophages around the dead cells within WAT (122). DP treatment led to WAT hypertrophy and dysfunction, in part by the inhibition of insulin signaling.

## Dibutyl Phthalate

Dibutyl phthalate (DBP) is a plasticizer found in plastic products such as toothbrushes, food wrappers, and in common household items as a fragrance-enhancing additive. DBP is a known EDC and obesogen that can affect fat accumulation and metabolic processes. DBP activates multiple receptors including the estrogen receptor, constitutive androstane receptor (CAR), the pregnane X receptor (PXR), and peroxisome proliferator-activated receptor subtypes (PPARα, -β, and -γ), which regulate the expression of genes encoding metabolic enzymes (126). Pregnant mouse dams were exposed to DBP from the 12th day of gestation to one week after birth. 4- to 5-month-old mice from the DBP group exhibited higher body weight, lower expression of UCP1, insulin insensitivity, greater endoplasmic reticulum stress, and levels of inguinal and epididymal WAT that were twice as high as controls (136). DBP exposure caused insulin resistance, suggesting the presence of a pre-diabetic condition which is often a comorbidity in obese individuals. Lower UCP1 levels impair the production of heat by thermogenic tissues and increased endoplasmic reticulum stress, greatly affecting mitochondrial function (136). Offspring of DBP-treated mice also showed significantly higher levels of binding immunoglobulin protein (Bip), and CCAAT/enhancer-binding protein homologous protein (Chop), which are two markers of ER stress that were associated with lower UCP1 levels. Offspring of DBP-treated mice appeared to be obesity prone *via* the inhibition of UCP1 caused by ER stress in adipose tissue after protein extraction and western blot analysis revealed that Bip and Chop were increased compared to controls (136).

## Tetrabromobisphenols

Brominated chemicals such as TBBPA are used in the production of plastics and electronics to reduce flammability as a safety precaution. However, TBBPA, along with its structural analogues TBBPA-MAE, TBBPA-MDBPE, TBBPA-BAE, AND TBBPA-BDBPE were shown to promote adipogenesis in 3T3-L1 cells by activating PPARγ and glucocorticoid receptors (137, 138), with TBBPA-MAE, and TBBPA-MDBPE acting as stronger promoters of adipogenesis than TBBPA itself (123). hMSCs were exposed to a mixture of TBBPA and TCDD (2,3,7,8-Tetrachlorodibenzo-p-dioxin) to mimic a more realistic scenario of chemical mixtures found in the natural environment. It was found that TBBPA alone predisposed hMSCs to differentiate into adipocytes and increased levels of PPARγ mRNA. In contrast, when TCDD was administered alone, it inhibited the differentiation process and PPARγ mRNA expression. However, when dually administered, TBBPA overrode TCDD’s inhibitory properties (124). Interestingly, TBBPA also increased the number of lipid droplets in hMSCs that differentiated into osteoblasts. While lipids in the bone marrow are an essential component of bone health, too many lipids can result in the impairment of stem and progenitor cell function (124, 125).

## β-Cypermethrin

β-Cypermethrin (βCYP) is a widely used pyrethroid, a class of chemicals commonly used as insecticides. Like other pyrethroids (synthetic relatives of the naturally occurring pesticide pyrethrin), βCYP is an EDC and has been found in humans. High concentrations of βCYP (concentrations of 25, 50, and 100 μM) promoted adipogenesis in 3T3-L1 cells (127). Mechanistically, β-CYP acts on 3T3-L1 by increasing the reactive oxygen species within cells *via* binding to the mitochondrial respiration chain complex 1. This disruption in the complex reduces the mitochondrial membrane potential, which is required for the browning of WAT, increased autophagy, and the miR-34a mediated polarization of macrophages to M2 cells. While He et al. reported a βCYP mediated downregulation of intracellular and extracellular miR-34a in 3T3-L1 cells, upregulation and increased expression of miR-34a has also been linked to obesity (139). The upregulation of miR-34 decreased SIRT1 expression, reduced NAMPT expression which in turn decreased NAD^+^ levels, which are crucial to cellular health, redox reactions, metabolism, and the browning of WAT (140, 141). NAMPT is important for the synthesis of NAD^+^ and is known to decrease in aging and obesity, leading to lower levels of NAD^+^ (139). Notably, an increase in NAD^+^ was also found in 3T3-L1 murine preadipocytes that differentiated into adipocytes after *in vitro* exposure to the obesogen, monoethylhexyl phthalate (142). It will be interesting to fully understand the role of miR-34a in obesity and how this is affected by pyrethroid expression.

## Metals

Heavy metals have been found to play a role in the disruption of the metabolic system including WAT, beige, and BAT imbalances. Cadmium (Cd) is a highly toxic heavy metal that can pollute water and food sources from burning fossil fuels and Cd is also present in tobacco and tobacco smoke [reviewed in (119)]. Cd is carcinogenic and proinflammatory, affecting multiple organs. Cd is a likely EDC that affects obesity and metabolism by altering adipose tissue through epigenetic changes [reviewed in 119]. *In vitro* exposure to cadmium altered the expression of MCP-1 (monocyte chemoattractant protein-1) in WAT, a chemokine for macrophage recruitment that plays a role in obesity and WAT inflammation [reviewed in (119, 120)]. Cd can bind to the estrogen receptor and it is also thought to bind to the androgen receptor, however, the exact mechanism through how Cd acts is unknown [reviewed in (119)].

Silver nanoparticles (AgNPs) have also become interesting as a potential EDC and obesogen. AgNPs are widely used as bactericides, and commonly found in the fabric of athletic clothing to eliminate odor. An exploration of the *in vivo* and *in vitro* role of silver nanoparticles in thermogenic beige adipocyte differentiation revealed that AgNP inhibited beige adipocyte differentiation, adipocyte thermogenesis, and mitochondrial functioning. AgNP elevated ROS levels within beige adipocytes and activated the MAPK-ERK signaling pathway which is involved in adipogenesis (114).

Arsenic is a metalloid that is widely found in the environment in soil, sediment, and water sources. Currently, more than 200 million people worldwide are exposed to drinking water containing dangerous levels of arsenic (143). Arsenic has been shown to affect adipogenesis in *in vivo* mouse models, as well as specifically target BAT (115). Arsenic exposure inhibited the differentiation of BAT and lowered expression of PPARγ, UCP1 and PGC1α, which are defining features of brown adipocytes (115). Arsenite, similarly reduced BAT differentiation, decreased mitochondrial functioning, and lowered thermogenesis in BAT by reducing UCP1 expression (117). Arsenite accumulates in BAT and suppressed Sestrin2 phosphorylation by ULK1, which under normal conditions, promotes the antioxidant effects of Sestrin2 against ROS that form within the cells (117, 118). Mice exposed to 300 μg/L of arsenite in drinking water for 9 weeks showed significant increases in inguinal WAT mass, lower thermogenic abilities when exposed to cold temperature, decreased beiging of WAT, and lowered expression of genes involved in regulating thermogenesis and metabolic functions (143).

## Sexual Dimorphisms

Multigenerational, transgenerational, and cohort based longitudinal studies offer a valuable platform to explore sex-dependent effects of EDCs and obesogens on males and females from birth into later life. Both human and mouse models have revealed obesogenic related differences in males and females, predisposing one or the other to an increased risk of overweight/obesity. A recent study found that BPA exhibited sex-dependent effects on mouse fetuses when given a low dose of 2.5 micrograms/kg/day. These mice showed changes in hepatic processes, including inflammatory effects and alterations in proteins involved in cholesterol and fatty acid functioning in females only (144). Human cohort studies revealed that prenatal BPA exposure increased the risk of obesity in young girls, but not in boys. A study done in rural East China showed that urinary BPA concentrations were linked to increased adiposity measures in girls at 7 years of age (145). Similarly, a study involving 585 high school aged students from Spain found that BPA disproportionately affected females over males, with an association between dietary BPA exposure and overweight/obesity in females (146).

The BPA analog BPS demonstrated sexually dimorphic effects after exposing female F0 mouse dams to an environmentally relevant dose of BPS in their drinking water during gestation through post-natal day 21, followed by a high fat diet fed to all offspring. F1 male mice became overweight, while F1 females developed dyslipidemia. The F2 generation showed increases in body weight and fat mass for both sexes, however, this generation was impacted the greatest in terms of sexual dimorphisms. Notably, F2 females showed a 35% increase in body weight over their counterpart males. Cholesterol levels, blood glucose levels, and circulating triglycerides were higher in males while non-esterified fatty acids were higher in females. Both sexes showed a downregulation in genes involved in lipogenesis, however, only F2 females downregulated genes involved in lipolysis. The F3 generation showed striking sexual dimorphism in that increased visceral adipose tissue mass only occurred in females, as did the upregulation of genes involved in lipogenesis (131).

When TBT (50 nM) was given to pregnant F0 mouse dams from conception throughout lactation *via* their drinking water, greater transgenerational and sex-dependent effects were observed in F4 male offspring (147). TBT was found to have largely male dependent transgenerational effects in the F3-F4 group, significantly increasing their body weight, fat storage, and creating changes in the male metabolome (147, 148). F4 Males developed a “thrifty phenotype” and had a reduced ability to mobilize fat storage after overnight fasting. Male mice that were exposed to TBT and later a high fat diet showed an increase in body fat that persisted even after the mice were returned to a normal chow (148).

Sex-dependent and adipocyte related epigenetic effects were observed in F3 offspring of F0 rats exposed to DDT *via* intraperitoneal injection during pregnancy (149). This study compared DMRs between vehicle control rats, lean rats exposed to atrazine, and DDT exposed rats. Adipocytes isolated from gonadal fat pads were used to identify unique DNA methylation regions between the three groups. Different DMRs were observed between males and females in the F3 generation of DDT exposed rats, indicating sex-dependent obesogenic effects in regions associated with obesity, T2D, and metabolic syndrome. A human longitudinal study revealed striking differences in 12-year-old males and females from a birth cohort study. Data from 240 children, who were exposed to DDT and its metabolite, DDE, *in utero*, were collected from the Center for the Health Assessment of Mothers and Children of Salinas (CHAMACOS). Maternal environmental DDT and DDE exposure was determined by collecting maternal serum during pregnancy. Data analysis was consistent with previous DDT and DDE studies, and revealed that *in utero* exposure increased obesity related measures for males only, indicating a sex-specific effect and risk for obesity in human males and not females (150).

Participants and data from the Hamamatsu Birth Cohort for Mothers and Children study were used to identify potential sex-specific prenatal exposure effects of perfluoroalkyl substances. Umbilical cord serum was measured for perfluorooctane sulfonate (PFOS) and PFOA concentration levels. BMI repeated measurements were taken from the children at ages 1, 4, 10, 18, 24, 32, 40, 50, and 66 months. The results indicated a significant difference in the BMI and body weight of girls. Prenatal exposure was linked to a lower birthweight in the girls, yet this effect was reversed as they aged, resulting in increased BMI and body weight as they reached 5 years of age (151).

## The Microbiome and Obesity

The gut microbiota is predominantly composed of bacteria from the phyla Bacteroidetes, Firmicutes, and Actinobacteria, which secrete enzymes that degrade dietary fiber (152). Individual differences in dietary habits can alter the diversity of microbial species within the intestinal tract (153–155). Moreover, the microbiome was found to differ markedly between normal weight and obese individuals. The obese microbiome was characterized by the predominance of Bacteroidetes over Firmicutes in the gut (155). Remarkably, transplant of the gut microbiome from obese mice to germ-free mice transferred the obese phenotype and microbiome and was characterized by a greater capacity to harvest energy (155). The gut microbiome is an important factor in energy harvest as it is a unique “organ” with microbial symbionts which help metabolize dietary polysaccharides and promote fat storage (156). Different individuals may share similar microbial genes which adapt to the unique metabolic needs of that individual, but have different bacteria lineages (157). Adult twins were observed to have similar bacterial communities than unrelated individuals, but there is no single bacterial phenotype, or “core” human gut microbiome defined by a set of microbial organismal lineages, shared by unrelated individuals. When samples of humanized mice (i.e. mice that received fecal microbiota transplantation of human feces) were fed the Western diet, they showed increased adiposity; a trait is transmissible *via* microbiota transplantation (158).

## The Role of EDCs in the Gut Microbiome

EDCs have been implicated in disease states such as insulin resistance, glucose intolerance, type 2 diabetes, and obesity (159), breast and prostate cancer (160, 161), as well as reproductive development disorders (162). In addition to their direct effects on physiology, exposure to EDCs, such as BPA, disturbs gut microbial composition (163). This may lead to changes in host lipid metabolism, among other effects (163). It was proposed that environmental obesogens could cause gut dysbiosis which might lead to inflammation and insulin resistance (164). The gut employs a multilayered mucus structure to maintain distance between the gut epithelial cells and the gut microbiota as a protective mechanism (165). EDCs could increase the permeability of the small intestine, increasing the likelihood that bacterial pathogens will enter the body’s circulation and target other organs (166). Food additives, such as artificial sweeteners (167, 168), and contaminants such as pesticide residues (169) can interfere with the gut microbiota and gut barrier function which could lead to intestinal, metabolic, and autoimmune disorders. Notably, obesity and T2D were found to be associated with intestinal dysbiosis (170) and gut barrier disruption (171). A list of potential and known obesogenic chemicals and how they affect the gut microbiome is presented in **Table 4**.

**Table 4:** Chemical obesogens and their effects on the microbiome.

Chemical	Source/Use	Proposed Mechanism	Effects	References
Fructose	Fruits, vegetables, and a natural sweetener in foods and beverages.	Decrease the expression of tight junction proteins, leading to increased intestinal permeability.	Increased intestinal permeability may allow endotoxins to diffuse through, causing chronic inflammation.	(172–174)
Artificial sweeteners or Non-Nutritive Sweeteners (NNS)	Sugar substitutes such as acesulfame potassium (Ace-K), saccharin, and sucralose, to name a few.	Decreased the relative abundance of *Clostridium*. Both sucralose and Ace-K decreased the abundance of *Akkermansia muciniphila*. Ace-K increased the synthesis of lipopolysaccharide (LPS) genes and may affect taste receptors.	NNS induced bacteriostatic effects and gut dysbiosis, leading glucose intolerance. Ace-K increased secretion of incretins, leading to weight gain. Increased LPS synthesis lead to inflammation.	(175–180)
Carbendazim (CBZ)	Agricultural fungicide and industrial preservative.	Changes in SCFA’s (short chain fatty acids) and resulted in decreased triglyceride levels. Decreased the abundance of Bacteroidetes and Verrucomicrobia and increased abundance of Actinobacteria.	Increased lipid absorption and inflammation which led to increased lipid stored as fat.	(181)
Chlorpyrifos (CPF)	Pesticide used on fruits and vegetables.	Chlorpyrifos causes microbial dysbiosis, causing in increase in *Streptococcus*, *Ruminiclostridium*, and *Parasutterella* and decrease in *Romboutsia*, *Turicibacter*, and *Clostridium.* Increased gut permeability due to decreased the expression of mRNA tight junction proteins.	Broken integrity of the gut barrier led to lipopolysaccharide entry and inflammation.	(165, 182–185)
Cadmium	Heavy metal ubiquitous in water, air, tobacco smoke, and plastics.	Low dose cadmium decreases diversity in early-life protective bacteria such as *Lactobacillus*, predisposing individuals to fat accumulation and obesity. Carbohydrate metabolizing bacteria such as *Bifidobacterium* and *Prevotella* are reduced.	Low-dose cadmium exposure caused increased fat accumulation and decreased bacterial diversity, especially in males.	(186, 187)
Bisphenol A (BPA)	Chemical used to make polycarbonate plastics and epoxy resins, including lining of food packaging.	Increased abundance of Proteobacteria and decreased the abundance of Bifidobacterium.	Acts in a sex-dependent manner, inducing pro-inflammation of gut microbiota primarily in females.	(170, 188, 189)
Polychlorinated Biphenyls (PCBs)	Ubiquitous chemical pollutants persistent in seafood and poultry due to their usage in dielectric and coolant fluids in the past.	Increased gut permeability, leading to increase the passage of pathogens and inflammation. Increased membrane disruptions in insulin-metabolizing murine fecal bacterium, thereby decreasing its fermentative ability.	Increased inflammation may lead to dysregulation of insulin signaling. Membrane disruptions in bacteria may cause gut dysbiosis.	(190–192)
Microplastic	Environmental pollutants commonly found in coastal oceans and terrestrial environments and includes BPA and phthalates.	Decreased mucus secretion leading to increased pathogen entry. Led to decrease of Verrumicrobia, Alphaproteobacteria, and *Oscillospira* and increase in *Parabacteroidetes*, *Prevotella, Dehalobacterium*, and *Bifidobacterium*.	May modify the gut microbiota and induce hepatic lipid disorder, particularly in male mice.	(193–196)

## Artificial Sweeteners

Non-nutritive sweeteners (NNS) are widely consumed dietary additives that could act as obesogens, impairing the growth of gut flora and inducing glucose intolerance (175). NNSs such as acesulfame potassium (Ace-K), saccharin, and sucralose induced bacteriostatic effects, which changed the composition of the gut microbiome (176) and induced glucose intolerance by promoting gut dysbiosis (177). Consumption of the NNS sucralose (a chlorinated derivative of sucrose) decreased the relative abundance of *Clostridium*, which converts primary bile acids into secondary bile acids (178). Both sucralose and Ace-K decreased the abundance of *Akkermansia muciniphila* (179), which is correlated with increased lipid metabolism and decreased inflammation (197). Sucralose may also affect taste receptors, thereby increasing the secretion of incretins glucose-dependent insulinotropic polypeptide (GIP) and GIP-1, leading to weight gain, hyperglycemia, hyperleptinemia, and hyperinsulinemia (180). Ace-K appears to have gender-specific effects as it decreased functional genes involved in energy metabolism in female mice but increased their expression in male mice (175). Ace-K also significantly increased the synthesis of lipopolysaccharide (LPS) genes, which may increase inflammation (175). Notwithstanding these results, the obesogenic properties of artificial sweeteners are highly disputed and future studies aimed at testing the links between artificial sweeteners, gut dysbiosis, and obesity will be required.

## Fructose Consumption

The first scientist to put fructose on the table as a cause for obesity was Dr. Robert Lustig in the early 2000’s. Fructose was found to cause changes in liver metabolism and energy signaling, creating a feedback loop in which insulin resistance and overeating occurs (198). Fructose is found in many processed food and beverages and is an obesogen known to cause glucose intolerance and insulin resistance (198). Consequently, it has been found that fructose restriction results in increased glucose tolerance and decreased insulin levels (199). On the other hand, fructose consumption causes hepatic *de novo* lipogenesis (DNL), which is dependent on the metabolism of fructose to acetate by the gut microbiota (200). The depletion of microbiota suppresses the conversion of fructose into hepatic acetyl CoA and fatty acids. It is proposed that there is a dual mechanism for fructose DNL: hepatic fructose metabolism promotes DNL transcriptionally while microbial acetate fuels DNL. Additionally, male mice fed a high-fat, high fructose diet (HFrD) demonstrated increased expression of inflammatory cytokines monocyte chemoattractant protein -1 (MCP-1_, toll-like receptor 4 (TLR4), interleukin-1 beta (IL-1β), and tumor necrosis factor alpha (TNF-α), which were associated with glucose intolerance and lipid accumulation (201). Fructose may cause gut dysbiosis and the upregulation of genes important for fat transport and storage such as CD36, fatty acid synthase (FAS), and sterol regulatory element-binding protein 1 (SREBP1), leading to hepatic steatosis (202). This may be a possible contributor to fatty liver disease (201). Exposure of rats to high-fat and sucrose (HFS) resulted in animals with 51% fat mass and 24% lean mass compared with 40% fat mass and 48% lean mass in the high-fat and fructose (HFF) diet group (203). Thus, while both types of diets increased fat mass, the HFS elicited substantially more fat mass than did the HFF diet. In addition to body fat mass composition, compared to the HFF group, the HFS group showed higher metabolic dysregulation and glucose intolerance, greater levels of liposaccharides and insulin, an increase in ROS as well as in markers for ROS, and an increase in lipid synthesis transcription factor, Srebp-1 (203). The comparison between fructose and sucrose indicate that the type of carbohydrate can have different effects, and that a HFS diet created more negative results than did a HFF diet. At the species level, there was a significant decrease in the abundance of *Coprococcus eutactus* in the HFS and HFF groups and an increase in abundance of *Lactobacillus reuteri* and *Bacteroides fragilis* in the HFF group. These two species have been associated with compromised integrity of the intestinal epithelium (203). Dysbiosis in the gut microbiota may also cause increased production of LPS, which stimulates a systematic inflammatory response (204) that may lead to insulin resistance and glucose intolerance (203). Rats fed a high fructose diet for one week showed reduced gut flora levels, increased inflammatory markers TNF-α and IFN-γ, increased glycation of gut proteins, and the reduced ability of extracted gut microbiota to grow on non-glycated proteins, likely causing the reduction in gut microbial survival (205). Interestingly, the glycation rate of high fructose fed rats was found to be higher than in diabetic rats (205).

It was proposed that one of the mechanisms through which fructose consumption causes obesity is by increasing intestinal permeability, leading to lipid accumulation, inflammation, and metabolic endotoxemia (a type of low-grade inflammation) (201). Fructose consumption has also been shown to affect the maternal microbiome during pregnancy, which is thought to alter the offspring’s gut health as well. The effects of fructose consumption on the maternal microbiome were tested by feeding rats 10% fructose *via* their water from 8 weeks through pregnancy. It was found that the maternal microbiome was altered to produce less beneficial bacteria, Lactobacillus and Bacteroides. Offspring maintained on fructose exposure showed a decrease in genes responsible for gut barrier function and a dysregulation of genes responsible for epithelial tight junction (172), which are diffusion barriers that regulate the passage of solutes across the epithelia (206). Shorter small intestines, lower birth weight, and increased fat mass were also observed, resulting in an overall unhealthy phenotype. In a non-high-fat diet-induced obesity model, microbiome dysbiosis was also associated with decreased expression of tight junction proteins (207). It is thought that the resulting increased gut permeability might allow endotoxins such as LPS to diffuse through the tight junctions and interact with host immune cells to cause chronic inflammation (173). More studies are needed to establish a direct link between obesogen-induced gut dysbiosis and intestinal permeability.

## Carbendazim

Carbendazim is a broad-spectrum, benzimidazole fungicide that is an EDC and an obesogen (181). Mice that were administered 0.1, 0.5, or 5mg/kg body weight per day carbenazim (CBZ) *via* their drinking water showed alterations in the gut microbiota that led to changes in the relative abundance of circulating short chain fatty acids (SCFAs) (181). Exposure to CBZ decreased the relative abundance of Bacteroidetes and Verrucomicrobia and increased the abundance of Actinobacteria, which resulted in altered levels of SCFAs (181). Microbial dysbiosis in the host resulted in downstream effects including decreased triglyceride (TG) synthesis in the liver, increased lipid absorption, and a multi-tissue inflammatory response (181). To compensate for the elevated lipid levels, the host reduced lipid synthesis in the liver and increased lipid storage in fat tissue (181). SCFAs and the gut microbiota work in conjunction to maintain optimal gut health, and the metabolite of SCFAs released by gut microbes play a role in epithelial cell health and intestinal barrier function. G-protein coupled receptors Gpr41 and Gpr43 have been discovered to work with SCFAs to mediate processes involved in host metabolism and intestinal epithelial functioning and health (181). Altered levels of SCFAs further change the expression of intestinal Gpr41 and Gpr43 as well as downregulates genes involved in host immune function (181). Taken together, these data are consistent with a model in which CBZ exposure caused gut dysbiosis that led to increased absorption of TG, leading to inflammation, hyperlipidemia, and increased fat storage. However, it is also possible that gut dysbiosis occurred in parallel with CBZ-altered lipid metabolism rather than causing it (181). The effects of CBZ have also been assessed in adult zebrafish who were exposed to 0, 30, and 100 μg/L of CBZ for 21 days (208). CBZ exposure altered the gut microbiota of the zebrafish, significantly decreasing phylum levels of Firmicutes, Bacteroidetes, Actinobacteria, α-Proteobacteria, γ-Proteobacteria and Verrucomicrobia (208).

## Chlorpyrifos

Chlorpyrifos (CPF) is a pesticide that is widely used on fruits and vegetables (182–184). CPF is also an EDC that decreased the levels of epinephrine, luteinizing hormone (LH), and follicle-stimulating hormone (FSH) (185), and induced gut microbial dysbiosis at low doses (209, 210). CPF exposure led to increased relative abundance of *Streptococcus*, *Ruminiclostridium*, and *Coriobacteriaceae* and decreased abundance of *Romboutsia*, *Turicibacter*, and *Clostridium* (185). CPF led to enriched abundance of *Parasutterella* in both normal fat diet fed and high fat diet fed rats; similar to results found after antibiotic treatment (185). CPF treatment decreased the abundance of SCFA-producing bacteria such as *Romboutsia*, *Turicibacter*, and *Clostridium* and enriched the pathogenic genus *Streptococcus*, which can result in an altered gut barrier (211). Interestingly, the effects of CPF on gut hormones appear to be age specific, as CPF increases the serum levels of glucagon-like peptide -1(GLP-1), pancreatic polypeptide (PP), peptide YY (PYY), and GIP in the newly weaned 3 week old rats in comparison to only stimulating the release of PPY and ghrelin when CPF is exposed during adulthood at 8 weeks of age up to 28 weeks of age (185). Zebrafish that were exposed to 30, 100, and 300 μg/L of CPF for 21 days, showed higher levels of oxidative stress coupled with the decrease of microbial diversity within the gut (74). Specifically, levels of *γ-Proteobacteria* showed a significant decrease in the gut microbiota, along with significant changes in 25 other types of genus level bacteria within the gut (74).

One of the proposed mechanisms through which CPF may cause obesity is by interfering with the gut microbiota and increasing gut permeability (185). CPF may increase gut permeability by reducing the expression of tight junction proteins (165). It was proposed that CPF breaks the integrity of the gut barrier by decreasing expression of mRNA encoding tight junction proteins which may lead to LPS entry and low-grade inflammation (165). Thus, the imbalance of microbiota, coupled with increased gut permeability, causes an increase in LPS and leads to inflammation (165), which are characteristics of obesity and diabetes. However, the mechanism(s) through which chlorpyrifos acts to modulate the microbiome and promote obesity are areas which needs further investigation.

## Cadmium

Cadmium is a heavy metal to which humans are readily exposed because it is ubiquitous in water, air, tobacco smoke, as well as plastics (186, 187). Exposure to cadmium during early life is especially detrimental since the gut microbiota is just being established (212). Cadmium decreases overall diversity in the gut microbiota, particularly in populations such as *Lactobacillus*, which are known to be protective early in life (213). This can reset the metabolic programming throughout life, leading to fat accumulation and obesity-related metabolic diseases (187). Low-dose cadmium exposure reduces the abundance of *Bifidobacterium* and *Prevotella*, which metabolize carbohydrates such as oligosaccharides and polysaccharides respectively (187, 214, 215). Interestingly, early life cadmium exposure elicited slightly more adiposity in males than in females (187, 212). However, the use of mostly male mice in metabolic studies of cadmium exposure (187) and metabolic variations between genders could contribute to these differences; thus, more research in this area is necessary (173). When three-week-old rats were administered cadmium at 0.1, 0.25, 1, or 4 mg/kg for eight weeks, changes of gut microbiota were observed (216). Mice in the 4 mg/kg group showed significant decreases in the beneficial genus *Prevotellaceae_NK3B31_group*, *Prevotella_9*, and *Lachnoclostridium*, and increases in *Escherichia_Shigella*, which increases oxidative stress and inflammation (216).

## Bisphenols

BPA is a chemical obesogen that is ubiquitous in the environment. BPA is used for making polycarbonate plastics and epoxy resins including those that line food packaging, as well as in thermal papers (170, 217). BPA is an EDC that may cause gut dysbiosis, metabolic disorders, and eventually lead to T2D (170, 188). Oral exposure to BPA dissolved in water in mice showed an increased the abundance of bacterial phylum Proteobacteria and a decrease in phylum Tenericutes (188). The increase in Proteobacteria, which is a microbial marker of dysbiosis, was caused by the induction of Epsilon proteobacteria (188). The increase of Epsilon proteobacteria was due to the increase of *Helicobacteraceae* which is associated with inflammatory bowel disease (218). Exposure to BPA caused a decrease in *Bifidobacterium*, which may be significant as some strains are known to have anti-inflammatory properties in intestinal epithelial cells (170, 219). Interestingly, the effects of BPA may be sex dependent as it induced a shift towards more pro-inflammatory gut microbiota in adult female mice and anti-inflammatory microbiota in adult male mice (189). Females injected with the antibiotic vancomycin did not experience BPA-induced gut dysbiosis, which suggests that gram-positive bacteria may not be a causal mechanism (189). BPA decreased the production of immunoglobulin A (IgA) which plays a key role in gut barrier integrity and gut homeostasis, leading to gut dysbiosis (170). Overall, the BPA microbiome had a similar species profile to one shaped by a high-fat and high-sucrose diet (188). These were linked to inflammatory bowel disease, metabolic disorders, and colorectal cancer (166). When 200 Crohn’s Disease (CD) patients were studied to assess the relationship between EDCs and CD, higher levels of BPA were found in patients with active CD compared to those in remission (220). In both remission and active CD groups, BPA was found to alter inflammatory responses in patients with gut barrier disruption and in those who tested positive for bacterial DNA in their blood (220). Taken together, it is evident that BPA could be playing an active role in the pathology of CD by disrupting the gut microbiome.

More recently, it has been shown that Bisphenol S (BPS) and Bisphenol F (BPF), which are BPA alternatives, cause microbial dysbiosis and are inversely related to developmental toxicity and estrogenic activity (221). Zebrafish that were exposed to BPF, BPS, or a combination of both chemicals at 1, 10, 100, or 1000 μg/L showed oxidative stress and intestinal inflammation as well as changes in their gut microbiome (222). Exposure to BPS and BPF, both separately and in conjunction, were found to increase pathogenic bacteria in the intestinal tract of the zebrafish, including the genus *Flavobacterium, Pseudomonas*, and *Stenotrophomonas* (222). In addition to direct action on the microbiome, exposure to EDCs such as BPA might affect the brain by disrupting the neural programming through the microbiome-gut-brain axis (223).

## Polychlorinated Biphenyls

PCBs (polychlorinated biphenyls) are ubiquitous environmental pollutants, and at environmentally relevant doses, may alter the abundance and diversity of the gut microbiome (224). PCBs may cause gut inflammation, which are implicated in metabolic diseases such as diabetes, through gut dysbiosis (190). Exposure to PCBs causes abdominal fat accumulation and obese individuals may be more vulnerable to PCB exposure than lean individuals (225). Male mice that were administered PCB *via* oral gavage had an increase in *Clostridiales* and decrease in *Bifidobacterium*, *Lactobacillus*, *Ruminococcus*, and *Oscillospira* in comparison to the controls. Decreases in *Bifidobacterium* and *Lactobacillus* are significant as these are probiotics that may reduce body weight, fat mass (226), and decrease endotoxemia (173). When exposed to a high dose of PCBs, there was an increase in *Prevotella* (227) which is associated with insulin resistance (228) and intestinal inflammation at mucosal sites (229), although *Prevotella* has been associated with beneficial health effects in another study (215). Additionally, when adult female mice were exposed to low-dose PCBs, there was an increase in *A. muciniphila* (227).

It is possible that PCB exposure may lead to obesity by disrupting the epithelial integrity and increasing gut permeability, rendering it vulnerable to pathogens in the gut mucosa (191). This may lead to high levels of inflammation, which is known to lead to dysregulation of insulin signaling, GLP-1 secretion from the intestine (190), and interfere with adipogenesis through the aryl hydrocarbon receptor (AhR) (230). Additionally, PCB exposure induced membrane disruptions in insulin-metabolizing murine fecal bacterium, decreasing its fermentative ability (192). Bacterial membrane disruptions were characterized by leakage of intracellular potassium, which is a central intracellular cation in bacterial cells (192). Therefore, bacterial membrane disruption may be a possible mechanism by which PCB exposure could cause gut dysbiosis (192). Recent studies have shown that PBC exposure induced deviations in the gut microbiome of mice, which further promoted non-alcoholic fatty liver disease in the animals. The obesity inducing receptor, PXR, and anti-obesity nuclear receptor, CAR, were knocked out in mice to assess the relationship between the receptors and the effects of PCB on metabolic functioning and the regulation of gut microbiome (231). It was previously shown that the PBC mixture, Aroclor1260, exacerbated NAFLD in mouse models *via* the activation of RXR and CAR pathways. It should be noted that mixtures of chemicals pose a challenge when studying the hormonal effects of individual chemicals, and conclusions cannot always be drawn from the effects of an individual chemical if it is a part of a mixture. Aroclor1260 was chosen as the test PCB mixture due to its relevance to human exposure, its resistance to metabolism, and its ability to bioaccumulate. PCB was further used to explore the role of PXR and CAR in relation to changes in the gut microbiome in diet induced obese mice and to determine what role PCR and CAR play in the gut-liver axis in relation to the gut microbiome. Both CAR and RXR knockout groups showed altered microbiome diversity, leading to greater hepatic and intestinal inflammation, dysregulations in energy metabolism, and nonalcoholic steatohepatitis. KO mice also showed a decrease in microbes related to lower inflammation levels and an increase in microbes related to inflammation, indicating that CAR and RXR may have a protective effect on the gut-liver-axis *via* the gut microbiome (231).

## Microplastic

Microplastics, which can range from nanometers to larger than five millimeters, are environmental pollutants commonly found in coastal oceans and terrestrial environments (193). They are particularly dangerous as they can persist in the marine ecosystem, rendering them difficult to remove (193). Moreover, many microplastics contain obesogens such as BPA (194) and phalates (195). Although there is a paucity of information regarding microplastics and their effects on the microbiome, a recent study showed that polystyrene microplastic may modify the gut microbiota and induce hepatic lipid disorder in male mice (196). Polystyrene microplastics caused decreased mucus secretion (196), which may allow pathogens to penetrate the intestinal mucosa. The relative abundance of Verrucomicrobia, Alphaproteobacteria, and *Oscillospira* decreased whereas the abundance of *Parabacteroidetes*, *Prevotella*, *Dehalobacterium*, and *Bifidobacterium* increased (196). While polystyrene microplastics were found to have a direct effect on the populations of various gut microbiota, it was also observed to disrupt lipid metabolism on a molecular level by altering the expression of key genes related to hepatic lipogenesis. Levels of PPARγ mRNA were found to decrease, while the mRNA levels of PPARα mRNA and fatty acid synthase (*Fas*), increased (196). PPARα is largely responsible for β-oxidation (196), and PPARγ is known to play key roles in adipocyte differentiation, lipid metabolism (232) and the storage and mobilization of lipids (233). It was proposed that polystyrene microplastics may cause gut dysbiosis and affect lipid metabolism in the liver through an indirect and unknown pathway related to the gut microbiota, although the mechanism remains unclear (196). More studies are necessary to validate whether microplastics lead to hepatic lipid metabolism disorder, and whether the effects are dependent or independent to the populations of gut microbiota.

## Conclusion

The study of obesogenic compounds is still in its early stages. However, the field has already shed ample light on factors that could contribute to the obesity pandemic beyond the energy balance paradigm. Metabolic disease has demanded increasing attention as the prevalence of worldwide obesity and related morbidities continues to rise (1). Thus far, ~50 obesogens have been recognized [reviewed in (30)], but more research must be done to discover those that remain unidentified and to test other candidate obesogens, *in vivo*. Reliable and replicable standardized detection assays and high throughput screening methods are needed to identify the remaining obesogens in the environment quickly and efficiently. The mechanisms of action through which known obesogens function by are still not well understood. *In vitro* studies (42) and *in vivo* studies (49) of TBT put the PPARγ and RXR pathways on the map as the major pathways of adipogenesis. Several obesogens have been found to function through the activation of this heterodimeric master regulator of adipogenesis. Other obesogens may function through pathways that remain to be identified. A more comprehensive understanding of obesogenic mechanisms and the mapping of their functional pathways will be key to implementing both preventative measures and therapeutic strategies against metabolic disease.

Current research on thermogenic fat, its function, and the role of its disruption in metabolic disease is calling for more attention on how modulating thermogenesis can be used in the treatment of metabolic diseases such as T2D and obesity. Increasing the activity of brown adipose tissue and promoting the beiging of WAT through genetic approaches, pharmacological methods, tissue-specific and cell type-specific strategies have become promising therapeutic avenues against obesity [reviewed in (234)]. There is a paucity of data concerning the role of obesogens and their effect on the microbiome, and more research is needed on how obesogens affect the gut. How the microbiome interacts with the gut-brain axis and how this influences appetite is a new field of research that may offer novel therapeutic strategies and directions for the treatment of obesity. The gut microbiota converts dietary nutrients into metabolites that can regulate appetite *via* vagal stimulation or through immune-neuroendocrine mechanisms (235). It is established that some obesogenic substances effect the gut microbiome, and when the gut microbiome moves away from its homeostatic levels, a variety of problems can arise, such as the dysregulation of orexigenic signals such as insulin and ghrelin which have effects on appetite and satiation (236). Potential therapeutics that target the gut microbiome, such as microbiome transplantation, may become viable future treatments for obesity.

The study of EDCs offers insights into how normal metabolic processes can be disrupted, and why the population is becoming unhealthier, particularly with respect to metabolic disease. As more MDCs are discovered, and more information is uncovered about currently used chemicals in industry, strategies to restrict usage and minimize exposure will become important. Avoidance of exposure through ingestion, inhalation, and direct contact is a definitive way to prevent metabolic disruption caused by EDCs before disease develops. *In vivo* transgenerational studies, which were only briefly discussed in this review, revealed epigenomic reprogramming effects and phenotypical metabolic effects caused by F0 chemical exposure on offspring into the F4 generation (147, 237). The existence of such “generational toxicity” demands further education about exposure prevention and transparency to keep the public and future generations safe from the effects of exposure to harmful chemicals.

## Author Contributions

BB, NM, CD, and CC contributed to writing the manuscript. BB and NM performed final editing, revisions, and submission.

## Funding

Supported by grants from the US National Institutes of Health, R01ES023316 and ES031139 to BB.

## Conflict of Interest

The authors declare that the research was conducted in the absence of any commercial or financial relationships that could be construed as a potential conflict of interest.

## Publisher’s Note

All claims expressed in this article are solely those of the authors and do not necessarily represent those of their affiliated organizations, or those of the publisher, the editors and the reviewers. Any product that may be evaluated in this article, or claim that may be made by its manufacturer, is not guaranteed or endorsed by the publisher.
